# The ubiquitin interacting motifs of USP37 act on the proximal Ub of a di-Ub chain to enhance catalytic efficiency

**DOI:** 10.1038/s41598-019-40815-z

**Published:** 2019-03-11

**Authors:** Noah Manczyk, Gianluca Veggiani, Joan Teyra, Amy W. Strilchuk, Sachdev S. Sidhu, Frank Sicheri

**Affiliations:** 1 Department of Biochemistry, University of Toronto, Toronto, ON M5S 1A8 Canada; 2 Lunenfeld-Tanenbaum Research Institute, Mount Sinai Hospital, Toronto, ON M5G 1X5 Canada; 3 Department of Molecular Genetics, University of Toronto, Toronto, ON M5S 1A8 Canada; 4 Banting and Best Department of Medical Research, Terrence Donnelly Center for Cellular and Biomolecular Research, University of Toronto, Toronto, ON M5S 3E1 Canada

## Abstract

USP37 is a deubiquitinase (DUB) with roles in the regulation of DNA damage repair and the cohesion of sister chromatids during mitosis. USP37 contains a unique insert of three ubiquitin interacting motifs (UIMs) within its catalytic DUB domain. We investigated the role of the three UIMs in the ability of USP37 to cleave di-ubiquitin chains. We found that the third UIM of USP37 recognizes the proximal ubiquitin moiety of K48 di-Ub to potentiate cleavage activity and posit that this mechanism of action may be generalizable to other chain types. In the case of K48-linked ubiquitin chains this potentiation stemmed largely from a dramatic increase in catalytic rate (k_cat_). We also developed and characterized three ubiquitin variant (UbV) inhibitors that selectively engage distinct binding sites in USP37. In addition to validating the deduced functional roles of the three UIMs in catalysis, the UbVs highlight a novel and effective means to selectively inhibit members of the difficult to drug DUB family.

## Introduction

Ubiquitination is the process whereby the small protein ubiquitin (Ub) is covalently attached to a substrate protein via a cascade of three (E1-E2-E3) enzymes. Multiple Ub moieties can be covalently linked together to form chains on the substrate via one of the seven lysine side chains or the free amino terminus of Ub. The eight Ub chain types can lead to a variety of cellular outcomes for a modified target protein, the best characterized of which is protein degradation through the 26S proteasome in the case of K48-linked chains^1^. Ubiquitination plays a key regulatory role in diverse cellular processes including DNA repair and cell-cycle progression, and as such the process is tightly regulated^2,3^.

The deconstruction of Ub chains is carried out by deubiquitinases (DUBs). In human, there are 7 families of DUBs based on domain structure, two of which have been recently discovered^4–7^, and among these, the Ub Specific Protease (USP) family represents the largest with more than 50 members. In contrast to the OTU family of DUBs, USP family members are notable for their generally poor ability to discriminate between different Ub chain linkages^8^. USPs commonly possess auxiliary domains, either N or C-terminal flanking, or internal to their catalytic domains at one of 5 possible insertion sites^9,10^. These domains function in different capacities, for example, binding Ub chains in the case of the UBA (Ub associated) domains of USP5, promoting protein complex formation in the case of the B-box domain in CYLD, or enhancing catalytic efficiency in the case of the DUSP-Ubl domains in USP4^11–13^.

USP37 is a USP family member implicated in the regulation of multiple critical cellular processes. For example, USP37 deubiquitinates cyclin A during G1 phase causing cyclin A stabilization and timely entry into S phase^14^. Additionally, USP37 facilitates the resolution of sister chromatids during prophase in a manner dependent on its DUB catalytic function^15^. In relation to its role maintaining chromosomal integrity, USP37 localizes to double-strand breaks and promotes BRCA1 inclusion into complexes responsible for homologous recombination^16^. Lastly, USP37 deubiquitinates and stabilizes the proto-oncogene c-Myc and the oncogenic fusion PLZF-RARA, suggesting that inhibition of USP37 DUB activity could have therapeutic potential^17,18^.

USP37 has a distinctive domain architecture consisting of an N-terminal PH domain, an interdomain linker and a C-terminal catalytic domain. Located within the catalytic domain is a large insertion of 284 amino acids (hsUSP37) containing three Ub-interacting motifs (UIMs) embedded at a site approximately 30 Å from the catalytic cleft. UIMs are single alpha-helical elements that bind to Ub with modest affinity (0.1–2 mM)^19^. UIMs conform to the consensus sequence e-e-x-x-φ-x-x-A-φ-x-(φ/e)-S-z-x-e, where e is an acidic residue, φ is a hydrophobic residue, z is a bulky hydrophobic or polar residue with high aliphatic content, A is alanine, S is serine and x is any residue^20^. UIM binding to Ub is routinely disabled by mutation of the consensus alanine position to glycine or the consensus serine position to alanine. In other DUBs, UIMs have been shown to confer cleavage preference for specific Ub chain types, such as K63-linked chains in the case of OTUD1 and Ataxin-3 or K48-linked Ub chains in the case of USP25^21–23^. Additionally, the UIMs of USP25 and Ataxin-3 have been shown to increase the ubiquitination state of the DUB itself, although the precise mechanism by which this is achieved remains unknown^24,25^.

Previous studies have shown that the UIMs of USP37 play an essential role in Ub cleavage activity and substrate binding properties of USP37. Specifically, A814G and S818A mutations to UIM2 and/or A836G and S840A mutations to UIM3 impaired the ability of USP37 to cleave K48- and K63-linked chains, while V712G and S716A mutations to UIM1 had no discernable effect^26^. Furthermore, while combined mutation of all three UIMs had a marked effect on DUB activity, it had no effect on the cleavage specificity of USP37 towards K48-linked chains over K63-linked chains^14,26^. Lastly, mutation of UIM2 and/or UIM3 perturbed USP37 binding to the cohesin regulator WAPL and to endogenous Ub-protein conjugates^15,26^. While these observations made clear that UIM2 and UIM3 play an important functional role in supporting the DUB activity of USP37, several questions remain unresolved, including: 1) do the individual UIMs impact the ability of USP37 to cleave the 6 Ub chain types not previously tested, 2) does mutation of the UIMs in USP37 selectively affect the k_cat_ or K_M_ of the enzyme, and 3) do the UIMs of USP37 act by engaging the proximal or distal position Ub moiety in a Ub chain. To address these questions, we have performed detailed mutational, biochemical and enzymatic characterization of the USP37 UIMs.

## Results

### Impact of UIMs on the Ub chain specificity of USP37

**Figure 1:**
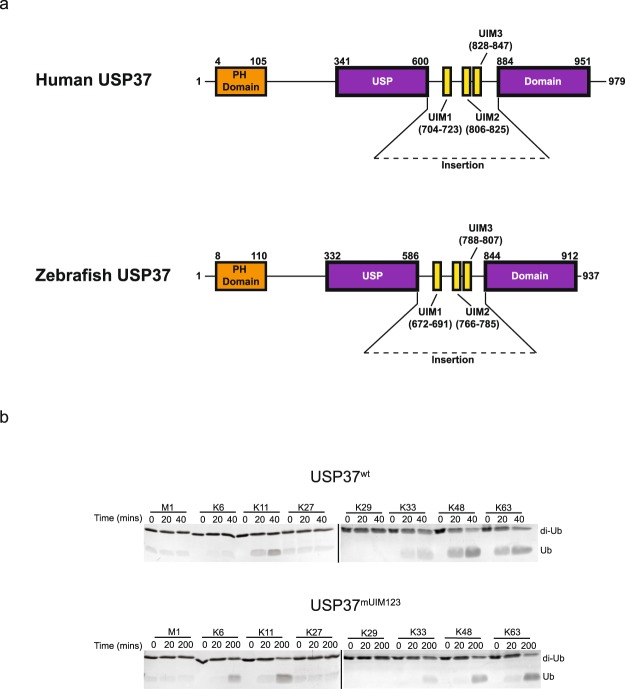
Di-Ub cleavage specificity of USP37^wt^ and USP37^mUIM1,2,3^. (**a**) Domain architecture of human and zebrafish USP37. (**b**) Deubiqutination time course for USP37^wt^ (top panel) and USP37^mUIM1,2,3^ (bottom panel) towards each of the 8 di-Ub linkages. Assay was performed in three independent experiments and representative gels are shown. Black lines delineate the separation of distinct gels. USP37^wt^ and USP37^mUIM1,2,3^ were used at a concentration of 1 nM. Uncropped versions of gels are shown in Supplementary Fig. S1.

We expressed the isolated catalytic domain of USP37 from *Danio rerio (*residues 312–927, denoted USP37^wt^), which unlike USP37 from human, mouse and chicken, could be expressed in bacteria in stable form for biochemical and enzymatic studies. *Danio rerio* and *Homo sapiens* USP37 share identical domain architectures and strong sequence similarity (72% and 77% similarity overall and over the catalytic domain, respectively) suggesting that this ortholog would serve as a good model for understanding the mechanism of action of the human enzyme (Fig. 1a). To determine comprehensively if the UIMs of USP37 contribute to chain linkage cleavage specificity, we tested the ability of USP37^wt^ to cleave all 8 possible di-Ub chain types including the 6 Ub chain types not previously tested. USP37^wt^ displayed a preference for K6-, 11-, 33-, 48- and 63-linked di-Ub chains (Fig. 1b, top panel). Strikingly, a mutant form of USP37 (USP37^mUIM1,2,3^) bearing mutations in all three UIMs predicted to abolish Ub binding (see methods for details) displayed no change in chain specificity. Indeed, USP37^mUIM1,2,3^ still displayed a strong preference for K6-, K11-, K33-, K48- and K63-linked di-Ub chains, although with reduced activity overall relative to USP37^wt^ (Fig. 1b, bottom panel).

We next performed experiments using tetra Ub chains and observed a similar overall trend (Supplementary Fig. S2). However, zebrafish USP37 displayed a greater preference for K48 over K63 chains, which more closely matched the reported chain specificity of the human enzyme^14,26^. Mutation of UIMs drastically decreased cleavage efficiency of USP37 against K48 and K63 tetra Ub chains without changing its preference for K48 tetra Ub chains. These findings further support the notion that the UIMs of USP37 do not confer specificity towards specific Ub chain types but are instead required for full enzymatic activity.

### The effect of mutations to all three UIMs on the kinetic parameters of USP37

**Figure 2:**
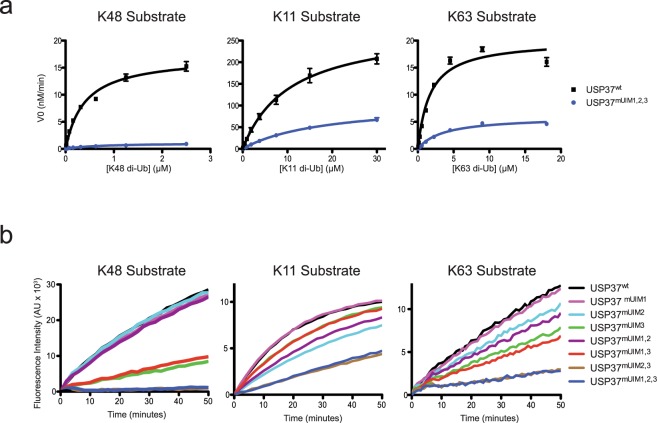
Activity of USP37^wt^ and USP37 mutants towards IQF K11, K48, and K63 probes. (**a**) Michaelis-Menten kinetic analysis of USP37^wt^ and USP37^mUIM1,2,3^ for the substrates K11-, K48- and K63-linked IQF di-Ubs. Curves represent measurements from three independent experiments each measured in duplicate. Values reported are mean ± SD. (**b**) Progress curves for USP37^wt^ and USP37 mutants towards IQF K11-, K48- and K63-linked di-Ubs. Assay was performed in duplicate in three independent experiments and representative curves are shown. For both experiments, USP37 was used at 25, 1.25 or 12.5 nM for K11, K48 or K63 di-Ub chains, respectively.

To determine how precisely the UIMs impact on the catalytic efficiency of USP37, we determined the effect of UIM mutations on the k_cat_ and K_M_ parameters of the enzyme. To this end, we performed kinetic experiments with USP37^wt^ and USP37^mUIM1,2,3^ against the three most preferred Ub chain substrates, namely K11-, 48-, and 63-linked chains, bearing internally quenched fluorescent (IQF) di-Ub probes (Fig. 2a). In this assay format, cleavage of di-Ub liberates the fluorophore from the quencher, yielding an increase in fluorescence proportional to the amount of substrate cleaved. To estimate how well the IQF substrate mimics the unmodified substrates, we performed a gel-based cleavage assay comparing USP37wt cleavage of unmodified K48 and K63 di-Ub with IQF labeled K48 and K63 di-Ub (Supplementary Fig. S3). We observed that IQF modifications adversely impacted the cleavage efficiency of substrate with the negative effect on K63 chains being more pronounced than K48 chains. As such, when using IQF substrates we restricted our comparisons to reactions using the same substrate type. For K48-linked chains, we observed a large reduction in k_cat_ (decreased ~15-fold) and a smaller perturbation in K_M_ (increased ~1.8-fold) (Fig. 2a left panel, Table 1 – see also Supplementary Fig. S4 for a zoom-in view of the USP37^mUIM1,2,3^ cleavage profile) in response to all three UIM mutations. For K11- and K63-linked chains, we observed more modest reductions in both k_cat_ (decreased ~2.6-fold for K11 and ~3.4-fold for K63) and K_M_ (decreased ~1.7-fold for K11 and ~2.1-fold for K63) (Fig. 2a middle and right panel, Table 1). Thus, the ability of UIMs to bind Ub plays differential roles on the kinetic parameters of USP37 depending on the Ub chain type tested.

**Table 1:** Michaelis-Menten analysis of USP37^wt^ and USP37^mUIM1,2,3^ for K11, K48 and K63 di-Ub substrates.

Substrate Enzyme	K11		K48		K63	
	USP37^wt^	USP37^mUIM1,2,3^	USP37^wt^	USP37^mUIM1,2,3^	USP37^wt^	USP37^mUIM1,2,3^
[E] (nM)	25	25	1.25	1.25	12.5	12.5
V_max_ (nM/min)	285 ± 22	108 ± 12	17 ± 2.1	1.1 ± 0.2	20 ± 1.2	6.0 ± 0.4
k_cat_ (s^−1^)	0.19	0.072	0.23	0.015	0.027	0.008
K_M_ (µM)	11 ± 2.3	19 ± 1.2	0.40 ± 0.09	0.72 ± 0.16	1.7 ± 0.08	3.6 ± 0.42

V_max_ and K_M_ values are mean ± SD.

### Effects of UIM mutations on the ability of USP37 to cleave K11-,K48- or K63-linked Ub chains

To determine if any particular UIM is strongly associated with cleavage of a specific chain linkage, we generated UIM mutations predicted to abolish Ub binding in all possible single, double and triple combinations. We then tested each protein for its ability to cleave K11-, K48- or K63-linked di-Ub chains. In characterizing the activity of the mutants against these 3 linkage types, we noted the following strong patterns of behavior (Fig. 2b).

UIM1 appeared completely dispensable for the proteolytic activity of USP37 against all tested Ub chain types. Specifically, introduction of disabling mutations within UIM1 had no adverse effect on enzyme activity regardless of the presence or absence of mutations in the other UIMs (compare enzyme pairs against the three di-Ub substrates: USP37^wt^ (black) to USP37^mUIM1^ (pink), USP37^mUIM2^ (cyan) to USP37^mUIM1,2^ (purple), USP37^mUIM3^ (green) to USP37^mUIM1,3^ (red) and USP37^mUIM2,3^ (brown) to USP37^mUIM1,2,3^ (blue)). These results are consistent with prior findings^26^, which tested UIM disabling mutations in the single (USP37^mUIM1^, USP37^mUIM2^, USP37^mUIM3^) and triple combinations (USP37^mUIM1,2,3^) in human USP37 against K48- and K63-linked poly-Ub chains and found no difference in cleavage activity between human USP37^wt^ and USP37^mUIM1^ towards K48- and K63-linked poly-Ub chains.

Both UIM2 and UIM3 appeared important for the ability of USP37 to cleave all Ub chain types but to varying degrees. In the case of K11- and K63-linked substrates, individually disabling UIM2 (USP37^mUIM2^) or UIM3 (USP37^mUIM3^) caused partial impairment of activity, while simultaneously disabling UIM2 and UIM3 (USP37^mUIM2,3^) resulted in an additive impairment of activity. These results are consistent with prior findings^26^, which showed that human USP37 with disabling mutations in all three UIMs had a larger decrease in activity towards K48- and K63-linked poly-Ub chains as compared to USP37 with disabling mutations in UIM2 or UIM3 alone.

In the case of the K48-linked Ub substrate, UIM3 was clearly more vital than UIM2 for the activity of USP37 (Fig. 2b). Disabling UIM3 alone (USP37^mUIM3^) resulted in a large decrease in activity, while disabling UIM2 alone (USP37^mUIM2^) had no observable effect on activity. However, the importance of UIM2 was revealed when UIM2 and UIM3 were disabled together, resulting in an enzyme (USP37^mUIM2,3^) in which activity was almost completely abolished relative to WT (~4% activity remaining based on V_0_). Such a drastic effect was not observed for the same double mutant when cleaving K11- or K63-linked chains, where USP37^mUIM2,3^ retained ~20% or ~18% of WT activity (based on V_0_), respectively. These results suggested that UIM1 does not contribute to the activity of USP37 towards K11-, K48- and K63-linked chains, whereas UIM2 and UIM3 do contribute to activity towards these chains, but to varying degrees that are dependent on the specific type of chain linkage. As the disabling mutations in the UIMs had the strongest effects on the ability of USP37 to cleave K48-linked Ub chains, we chose to focus subsequent functional characterizations on this substrate type.

### Interrogation of proximal or distal Ub binding by the UIMs of USP37

**Figure 3:**
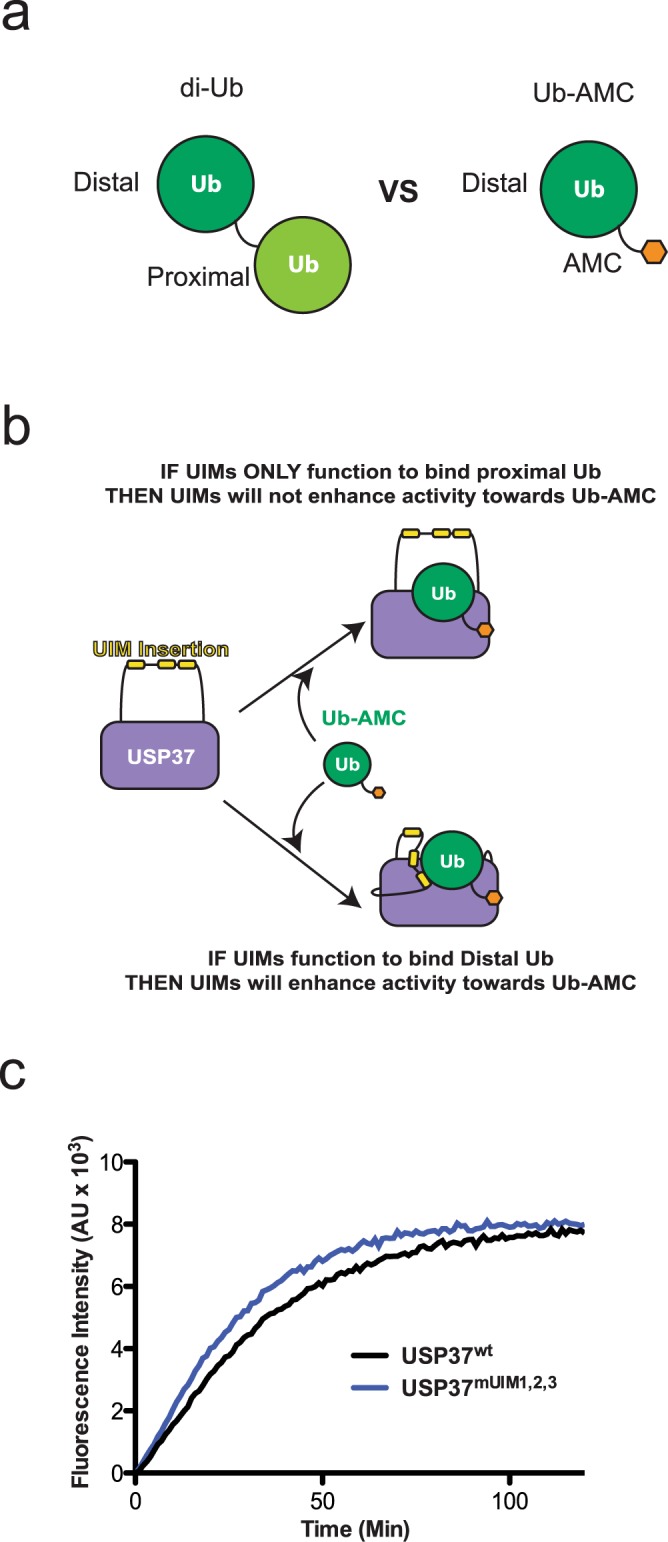
USP37^wt^ and USP37^mUIM1,2,3^ activity towards Ub-AMC. (**a**) Cartoon models of di-Ub and Ub-AMC illustrating how di-Ub contains both a distal and proximal Ub, while Ub-AMC contains only a distal Ub. (**b**) Cartoon model of the two possibilities of how the UIMs of USP37 can interact with Ub-AMC and the expected effects of each on Ub-AMC hydrolysis. The cartoon depicts a model in which the UIMs bind to the same di-Ub chain displayed across the enzyme active site. (**c**) Progress curve for USP37^wt^ and USP37^mUIM1,2,3^ activity against Ub-AMC. Assays were performed in duplicate in three independent experiments and representative curves are shown. Enzymes were used at a concentration of 0.5 nM.

To determine if the UIMs of USP37 specifically engage the proximal Ub (primary amine donating) or the distal Ub (carboxy terminus donating) in a K48 di-Ub chain substrate to exert their effect on cleavage activity, we first made use of the model substrate Ub-AMC in which the proximal Ub moiety is replaced with a cleavable fluorescent molecule (AMC, 7-Amino-4-Methylcoumarin) (Fig. 3a).

The proximal Ub is absent in Ub-AMC, and thus, we posited that if the UIMs act on the proximal Ub, we would observe no difference in cleavage by USP37^wt^ or USP37^mUIM1,2,3^ (Fig. 3b). In contrast, if the UIMs act by binding to the distal Ub moiety of a di-Ub substrate, then we would expect a major difference in the cleavage of Ub-AMC by USP37^wt^ or USP37^mUIM1,2,3^, as the distal Ub is fully available for binding (Fig. 3b). In comparing the activity of USP37^wt^ and USP37^mUIM1,2,3^ against Ub-AMC, we observed no difference in DUB activity (Fig. 3c). This result is consistent with the UIMs of USP37 acting by engaging the proximal Ub (Fig. 3c).

**Figure 4:**
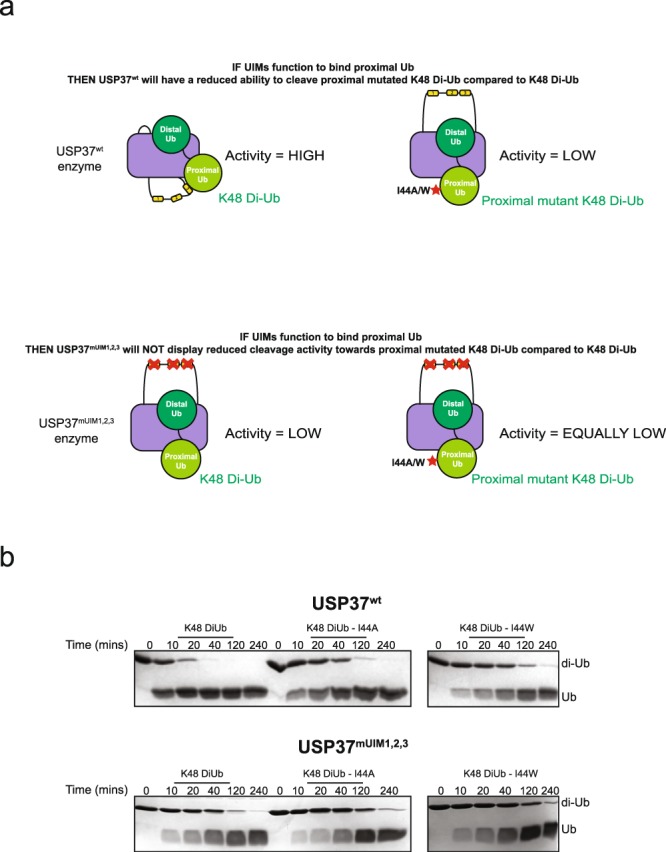
USP37^wt^ and USP37^mUIM1,2,3^ activity towards proximally mutated K48 di-Ub. (**a**) Cartoon model for how USP37^wt^ and USP37^mUIM1,2,3^ are expected to cleave K48 and proximally mutated K48 chains. The cartoon depicts a model in which the UIMs bind to the same di-Ub chain displayed across the enzyme active site. (**b**) Deubiquitination time course for USP37^wt^ (top panel) and USP37^mUIM1,2,3^ (bottom panels) towards K48, K48^I44A^, and K48^I44W^ di-Ub. Assay was performed in three independent experiments and representative gels are shown. USP37 enzymes were used at a concentration of 5 nM. Black boxes delineate distinct gels or cropped areas of same gel. Uncropped versions of gels are shown in Supplementary Fig. S1.

To further verify that the UIMs of USP37 act by engaging the proximal Ub of K48 di-Ub, we used a mutant form of K48-linked di-Ub chains in which substitutions were incorporated into the proximal Ub moiety at position 44 (I44A or I44W). The Ile44 side chain of Ub comprises part of the canonical hydrophobic binding surface for UIMs, and substitutions at this position are likely to perturb UIM binding. If the UIMs of USP37 bind to the proximal Ub of a di-Ub substrate, then di-Ub proteins with substitutions at position 44 of the proximal Ub should be poorer substrates relative to WT di-Ub, in the case of the USP37^wt^ but not in the case of USP37^mUIM1,2,3^ in which all UIMs are disabled (Fig. 4a). Indeed this is what we observed. Compared with cleavage of WT K48-linked di-Ub, USP37^wt^ displayed a reduced ability to cleave di-Ub substrates containing an I44A/W substitution (Fig. 4b top panel). In contrast, USP37^mUIM1,2,3^ displayed similar activity for cleavage of all three substrates, albeit at reduced rates relative to the WT enzyme and substrate forms (Fig. 4b, bottom panel). We also tested the three single site UIM mutants of USP37 for their ability to cleave K48 di-Ub substrates bearing the I44A/W substitution in the proximal Ub position. All three single site mutant proteins displayed a reduced ability to cleave the mutant substrates relative to wild type K48 di-Ub, similar to the behavior of USP37^wt^. This expected result is consistent with a degree of functional redundancy between UIMs (Supplementary Fig. S5). Together, these results indicate that the UIMs of USP37 act to support enzymatic activity by selectively engaging the proximal Ub of K48 di-Ub.

### Interrogation of UIM function with Ub Variants

Ub variants (UbVs) are engineered forms of Ub that bind specifically and tightly to a target protein of interest^27^. A UbV usually binds with higher affinity to preexisting Ub-binding site in a manner similar to the native interaction^27–30^. UbV reagents have been used previously to interrogate the mechanism of action and biological function of Ub-related enzymes both *in vitro* and *in vivo*^27,29–36^.

To further verify our model that UIM2 and UIM3 act by selectively binding to the proximal Ub of K48 di-Ub, we used phage display to derive one UbV that bound to the core catalytic domain lacking the insert containing the UIMs (UbV.core), another UbV that bound to the first UIM domain of USP37 (UbV.UIM1), and a third UbV (UbV.UIM*) that bound all three the UIM domains of the enzyme (Supplementary Fig. S6). We assessed the binding specificity of each UbV using GST/MBP pulldown experiments with the UbVs as prey and each isolated UIM (His-MBP-UIM1, His-MBP-UIM2, His-MBP-UIM3), the catalytic domain (GST-USP37) or the catalytic domain lacking all three UIMs (GST-USP37^Δloop^) as bait (Supplementary Fig. S7). We also assessed the binding of the three UbVs with human USP37 and observed coincidentally that UbV.UIM* and UbV.UIM1 but not UbV.core were retained by GST-hUSP37 in pulldowns (Supplementary Fig. S8).

**Figure 5:**
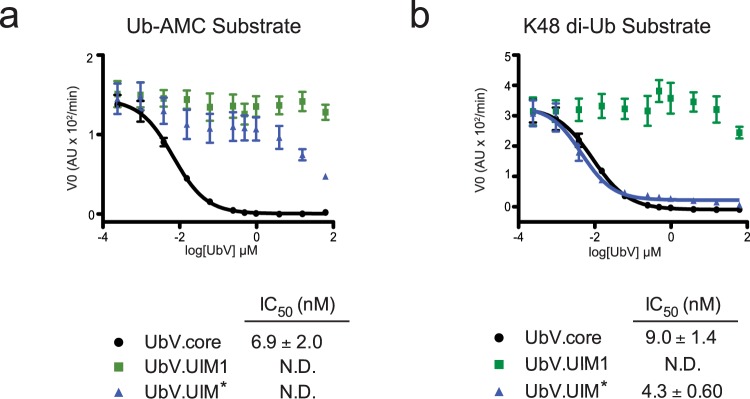
Analysis of inhibitory effects of UbVs on USP37 activity. (**a**) Inhibition of USP37^wt^ cleavage of Ub-AMC by UbV.core, UbV.UIM1, and UbV.UIM*. Curves represents measurements from three independent experiments. Values reported are mean ± SD. (**b**) Inhibition of USP37^wt^ cleavage of IQF K48-linked di-Ub by UbV.core, UbV.UIM1 and UbV.UIM*. Curves represents measurements from three independent experiments. Values reported are mean ± SD. For both experiments USP37 was used at a concentration of 0.5 nM.

We next determined the ability of each of the three UbVs to inhibit hydrolysis of Ub-AMC by zebrafish USP37^wt^. Mutation of UIM1 had no effect on enzyme function, and as expected, UbV.UIM1 did not affect USP37^wt^ activity (Fig. 5a). In our working model, UIM2 and UIM3 engage the proximal Ub moiety of K48 di-Ub to influence catalytic activity. As Ub-AMC lacks a proximal Ub, we were not surprised to see that UbV.UIM* had little effect on hydrolysis of this substrate (Fig. 5a). In contrast UbV.core potently inhibited Ub-AMC cleavage (IC_50_ ~9 nM). This result is consistent with the likelihood that UbV.core directly competed for binding to the distal Ub-binding site of USP37 (Fig. 5a).

We next determined the ability of each of the three UbVs to inhibit the activity of zebrafish USP37 for cleavage of the K48-linked di-Ub substrate bearing an internally quenched fluorophore (IQF). UbV.UIM1 had no effect on this activity (Fig. 5b), consistent with our mutational data for UIM1 (Fig. 2b). In contrast, UbV.UIM* potently inhibited cleavage of the K48-linked di-Ub substrate, as did UbV.core (Fig. 5b). Taken together, these results support our model in which UIM2 and UIM3 engage the proximal Ub of K48 di-Ub to enhance cleavage by USP37, whereas UIM1 is not involved in substrate recognition.

## Discussion

Our results are consistent with a model whereby UIM2 and UIM3 of USP37 bind the proximal Ub of K48 di-Ub to increase catalytic efficiency. In the case of K48-linked substrates, binding of the UIMs to the proximal Ub increased catalytic efficiency of USP37 largely through k_cat_ and to a lesser degree through K_M_. This result was somewhat counter-intuitive since we expected that binding of the UIMs of USP37 to Ub to have a more pronounced effect on K_M_ (a proxy for binding affinity) since others have shown that UIM mutations perturbed the ability to pull down Ub conjugates^26^. Furthermore, we observed that in the case of K48-linked di-Ub substrate, UIM3 was more vital than UIM2 for the proteolytic activity of USP37. This behavior was not noted in the same previous study^26^. A possible explanation for our findings relates to the main type of Ub conjugates investigated, which involved chains of variable length greater than 2 moieties^26^ versus Ub chains of fixed length equal to 2 Ub moieties used here. Characterizing the kinetic parameters of cleavage of longer Ub chains by USP37 could help to address this point. The issue of how the binding of the UIMs of USP37 to the proximal Ub of K48 di-Ub enhances activity remains an open question. We envision a model whereby the UIMs optimally position a di-Ub chain across the active site of the USP domain for more efficient catalysis of the inter-Ub isopeptide bond by selectively binding the proximal Ub position. We expect the UIMs to act upon the same substrate engaged by the USP domain active site (i.e. in cis) as zebrafish USP37 displays no tendency to multimerize in solution as assessed by size exclusion chromatography (Supplementary Fig. S9). Whether this model is generalizable to other Ub chain types, requires further verification. Furthermore, as our experiments were largely focused on di-Ub substrates, it is possible that the UIMs could function in additional capacities for longer chains types. A co-structure of USP37 with a Ub chain substrate would be highly informative for resolving the remaining mechanistic questions.

We developed UbVs that bound the core catalytic domain or the UIMs of USP37 and functioned as potent inhibitors of catalytic activity *in vitro*. The UbVs targeting the UIMs represent the first report of UbV DUB inhibitors that act by targeting domains distinct from the catalytic domain. Notably, UbVs have been shown to inhibit DUBs in cells^27^, and we posit that the UbVs developed here may prove useful as reagents for future studies interrogating the *in vitro* biochemical functions and *in vivo* biological functions of USP37.

While inhibitors of USP enzymes have been developed, specificity has been a recurring problem^37^. This may be due to the lack of differentiating features between the catalytic sites of USP family members. Only recently have groups demonstrated effective specific inhibitors against a USP enzyme, namely USP7^38–41^. Interestingly, one of these inhibitors did not target the conserved active site directly, but rather, acted by targeting a USP7 loop distinct from other USP family members^39^. In an analogous manner, we exploited the unique UIMs of USP37 to derive a specific inhibitor of USP37.

As noted, USP37 stabilizes the proto-oncogene c-MYC and the oncogenic fusion PLZF/RARA in cells^17,18^. The demonstration that knockdown of USP37 decreases the stability of these oncogenic proteins, raises the possibility that specific and potent inhibitors of USP37 may provide a viable therapeutic strategy to treat human cancers. To this end, targeting UIM2 and UIM3 may provide the basis for potent and specific inhibition of USP37 DUB activity.

## Materials and Methods

### Protein Purification

For biochemical experiments, a codon optimized gene (GeneArt) encoding for *Danio rerio* USP37^wt^ (312–927) containing a N-terminal GST tag was expressed in *Escherichia coli* BL21(DE3)-RIL cells. Cells were lysed by homogenization in lysis buffer (50 mM HEPES pH 8, 500 mM NaCl, 5% glycerol, 5 mM DTT) with 0.5 mM phenylmethane sulfonyl fluoride (PMSF). After clarification via centrifugation, supernatant was incubated via gravity column with glutathione resin and washed with lysis buffer. Protein bound to glutathione resin was incubated overnight in presence of TEV protease at 4 °C. Protein was eluted with lysis buffer and concentrated to ~4 mg/mL and subsequently flash diluted to ~0.2 mg/mL in low salt buffer (25 mM HEPES pH 8, 50 mM NaCl, 5 mM DTT) and loaded onto an HiTrap Q HP anion exchange column (GE Healthcare). Protein was eluted via a linear gradient with low and high salt buffer (25 mM HEPES pH 8, 500 mM NaCl, 5 mM DTT). Fractions containing USP37^wt^ were pooled, concentrated and injected onto a Superdex 200 column, pre-equilibrated with sizing buffer (25 mM HEPES pH 8, 150 mM NaCl, 5 mM DTT). Fractions containing USP37^wt^ were pooled, concentrated and stored at −80 °C. USP37 mutants (V680G and S684A for UIM1, A774G and S778A for UIM2, and A796G and S800A for UIM3) were cloned from the WT construct using the QuikChange method (Stratagene) and expressed and purified in similar fashion.

UbVs containing a 6xHis (UbV.UIM1), a 6xHis-GST (UbV.core) and 6xHis-MBP (UbV.UIM*) tag were expressed in *E*. *coli* BL21(DE3)-RIL cells. Cells were lysed by sonication in low imidazole buffer (50 mM HEPES pH 7.5, 500 mM NaCl, 5% glycerol, 5 mM imidazole, 4 mM β-mercaptoethanol (β-mer), 0.5 mM PMSF). After clarification via centrifugation, supernatant was loaded onto a Hitrap IMAC HP column (GE Healthcare). Protein was eluted using a linear gradient (5–300 mM imidazole) and dialyzed overnight in low imidazole buffer in the presence of TEV protease. A subtractive nickel column affinity purification was performed to remove TEV protease. Flow-through was collected, concentrated and injected onto a Superdex 75 column pre-equilibrated with sizing buffer (25 mM HEPES pH 7.5, 100 mM NaCl, 2 mM DTT). Protein fractions were pooled, concentrated and stored at −80 °C.

For phage display selections, USP37 protein fragments were expressed either as a GST-fusion (USP37^Δloop^ (*Danio rerio* 312–927 Δ612–841)), His-GST-fusion (human UIM2 (806–825)) or His-MBP fusion (UIM1 (*Danio rerio* 670–693)). Purification of GST-USP37^core^ followed the same strategy as USP37^wt^ with the exception that GST-USP37^core^ was eluted from glutathione resin using lysis buffer with 10 mM reduced glutathione rather than by cleavage with TEV protease. His-GST-UIM2, cells were lysed by sonication in low imidazole buffer (50 mM HEPES pH 7.5, 500 mM NaCl, 10 mM imidazole, 20 U/ml Benzonase (EMD Millipore), protease inhibitor cocktail (Sigma-Aldrich)). After clarification by centrifugation, supernatant was incubated on Ni-NTA resin (Qiagen). Protein was eluted with an imidazole buffer gradient (30–300 mM), fractions containing UIM2 were pooled, and the buffer was exchanged by dialysis into PBS pH 7.4 at 4 °C. The protein was then concentrated and stored at −80 °C. Protein was concentrated and stored at −80 °C. His-MBP-UIM1, cells were lysed by sonication in low imidazole buffer (50 mM HEPES pH 7.5, 500 mM NaCl, 5 mM imidazole, 5% glycerol, 4 mM β-mer, 0.5 mM PMSF). After clarification by centrifugation, supernatant was loaded onto a Hitrap IMAC HP column (GE Healthcare). His-MBP-UIM1 was eluted using a linear imidazole gradient (5–300 mM). Protein was dialyzed overnight into PBS pH 7.4 at 4 °C, concentrated and stored at −80 °C.

### Size-exclusion chromatography

USP37^wt^ was purified by glutathione resin and anion exchange chromatography as detailed in the *protein purification* methods section. USP37wt was concentrated and injected (1 mL of ~2 mg/mL) onto a Superdex S200 (GE Healthcare) previously equilibrated with 25 mM HEPES pH 8, 150 mM NaCl, 5 mM DTT. Absorbance at 280 nm was used to monitor the elution of the protein and determination of effective molecular weight was performed using a formula derived from protein standards.

### Gel-based deubiqutination assays

Gel-based deubiquitination assays were performed in 25 mM Hepes pH 8, 150 mM NaCl, 5 mM DTT, 0.1 mg/ml BSA. For data in Fig. 1, 1 nM USP37 enzyme was incubated with 9 µM di-Ub substrate at 20 °C. Di-Ub chains were enzymatically produced by our group (K11, K48, and K63) or Boston Biochem (K6, K27, K29, and K33), with the exception of Met1 di-Ub chains, which were produced as a tandem fusion directly in *E*. *coli*. Met1-linked Ub chains were produced according to published methods^42^. K48- and K63-linked Ub chains were produced as described^43^, with the use of a K48R and K63R Ub mutant (for distal Ub) and with the variation of using a Ub with the last two Gly residues deleted (Ub∆GG, for the proximal Ub). K11-linked Ub chains were produced similarly (K11R Ub for distal Ub and Ub∆GG for proximal Ub), with the exception of using 30 µM Ube2S as the E2 conjugating enzyme according to published methods^44^. K6-, K27-, K29- and K33-linked Ub chains were obtained from a commercial source (Boston Biochem, catalog #: UC-11B, UC-61B, UC-81B, and UC-101B, respectively). Time points were analyzed by SDS-PAGE and staining with gel-code blue (Thermofisher, catalog # 24590). Images were taken on a Bio-Rad ChemiDoc MP Imaging System. Images were cropped using Image Lab (Bio-Rad).

For data in Fig. 4b and Supplementary Fig. S5, 5 nM USP37 enzyme was incubated with 18 µM di-Ub substrate at 20 °C. Mutant K48-linked di-Ubs were produced with the same method as WT K48-linked di-Ub using K48R Ub (for distal Ub) and I44A/W Ub∆GG (for proximal Ub). Time points were analyzed by SDS-PAGE and staining with Coomassie blue. Images were taken on a Bio-Rad ChemiDoc MP Imaging System. Images were cropped using Image Lab (Bio-Rad).

For data in Supplementary Fig. S2, 10 nM USP37 enzyme was incubated with 6 µM tetra-Ub at 20 °C. K48 and K63 tetra-Ub chains were obtained from a commercial source (Boston Biochem, catalog #: UC-210B and UC-310B, respectively). Time points were analyzed by SDS-PAGE and staining with Coomassie blue. Images were taken on a Bio-Rad ChemiDoc MP Imaging System. Images were cropped using Image Lab (Bio-Rad).

For data in Supplementary Fig. S3, 1 nM USP37 enzyme was incubated with 9 µM of the indicated di-Ub substrate at 20 °C. Time points were analyzed by SDS-PAGE. Gels were fluorescently scanned using a Bio-Rad ChemiDoc MP Imaging System and Green Epi illumination with a 605/50 filter. Gels were then stained with Coomassie blue and imaged on a Bio-Rad ChemiDoc MP Imaging System. Images were cropped using Image Lab (Bio-Rad).

### Fluorescence deubiquitination assays

Fluorescent deubiquitination assays were performed at 30 °C in 20 µL DUB buffer (25 mM Hepes pH 8, 150 mM NaCl, 5 mM DTT, 0.1 mg/ml BSA, 0.03% BRIJ-35) in 384-well black flat-bottom plates (Corning 3573). Measurements were taken each minute in a BioTek Synergy Neo plate reader machine using excitation and emission wavelengths of 540 and 580 nM respectively for assays using internally-quench-fluorescent (IQF) probes and excitation and emission wavelengths of 345 and 445 nM respectively for assays using Ub-AMC.

For data in Fig. 2a, substrate protein was a mixture of 1 part IQF K11- (BostonBiochem, catalog #: UF-440), K48- (Lifesensors, catalog # DU4802) or K63-linked (Lifesensors, catalog # DU6303) di-Ub and 4 parts of the respective unlabeled chain. 25 nM USP37 (wild type or mutant form) was added to the indicated concentrations of K11 substrate. 1.25 nM USP37 enzyme was added to the indicated concentrations of K48 substrate. 12.5 nM USP37 enzyme was added to the indicated concentrations of K63 substrate. V_0_ was calculated from the linear portion of the progress curve. V_0_ was converted from fluorescent units/min to µM/min using a Ub-TAMRA standard curve. V_0_ was plotted against di-Ub concentration and kinetic parameters (K_M_ and k_cat_) were calculated using GraphPad Prism 5.

For data in Fig. 2b, 25 nM (for K11), 1.25 nM (for K48) or 12.5 nM for (K63) of the indicated USP37 proteins were mixed with 250 nM of IQF K11, K48 or K63 di-Ub chains. For data in Fig. 3c, 0.5 nM USP37^wt^ or USP37^mUIM1,2,3^ was incubated with 250 nM Ub-AMC (Boston Biochem, catalog # U-550). For data in Fig. 5, 1 nM USP37^wt^ (10 µL) was incubated with 5 µL of varying concentrations of the respective UbV (0.008–250 µM) for 20 mins at 20 °C. 1 µM Ub-AMC or IQF K48 (5 µL) was then added, and measurements were taken. V_0_ was calculated after initial noise dissipated and linear portion of the progress curve appeared. V_0_ was plotted against UbV concentration and IC_50_ values were calculated using GraphPad Prism 5.

### Pulldown assays

GST-USP37 (*Danio rerio* 312–927), GST-USP37^Δloop^ (*Danio rerio* 312–927 Δ600–884), and GST-hsUSP37 (*Homo sapiens* 332–969) were expressed in *E*. *coli* BL21(DE3)-RIL cells that were lysed by homogenization in lysis buffer (25 mM Hepes pH 8, 500 mM NaCl, 5% Glycerol, 5 mM DTT), whereas GST, HisMBP, HisMBP-UIM1 (*Danio rerio* 670–693), HisMBP-UIM2 (*Danio rerio* 764–787) and HisMBP-UIM3 (*Danio rerio* 786–807) were expressed in *E*. *coli* BL21(DE3)-RIL that were lysed by sonication in lysis buffer. Proteins were incubated with their respective affinity resin (Glutathione or Maltose) for 1 hour for batch binding. Unbound proteins were washed away with lysis buffer. Beads were then equilibrated in binding buffer (25 mM Hepes pH 8, 150 mM NaCl, 5 mM DTT, 0.1 mg/ml BSA, 0.03% BRIJ-35). 30 µg of UbV.core, UbV.UIM1 or UbV.UIM* were then incubated with the beads for 1 hour and washed twice with binding buffer (wash volume = 25 times bed volumes). Samples were resolved and analyzed via SDS-PAGE with Coomassie blue staining. Images were taken on a Bio-Rad ChemiDoc MP Imaging System.

### Selection of UbVs by phage display

The phage displayed UbV library 2 (Ernst *et al*.^27^) was used to perform binding selections for USP37^Δloop^ and UIM1. For selection of UbVs for binding to UIM2, we used a new library (Veggiani and Sidhu, unpublished results) that was designed by inspection of the structure of a UbV bound to a yeast UIM (Manczyk *et al*.^28^). Selections were performed as described (Ernst *et al*.^27^). Briefly, library phage pools were cycled through rounds of binding selections with target proteins immobilized on 96-well Maxisorp Immunoplates (Nunc). After five rounds, phage from individual colonies were assessed for binding to immobilized proteins by phage ELISA (Persson *et al*., 2013), and clones that bound to target protein but not to negative control proteins were subjected to DNA sequencing to decode the sequences of the displayed UbVs.

## Supplementary information

Supplementary Information

## Data Availability

Data generated or analyzed during this study is included in this published article or is available from the corresponding authors upon reasonable request.
