# Supplementary material for: The ubiquitin interacting motifs of USP37 act on the proximal Ub of a di-Ub chain to enhance catalytic efficiency
Source: Sci Rep. 2019 Mar 11;9:4119. doi: 10.1038/s41598-019-40815-z (PMC6412040; doi:10.1038/s41598-019-40815-z)
Supplement: Supplementary file 1 — Supplementary Information [file 41598_2019_40815_MOESM1_ESM.pdf]

Supplementary Information

The ubiquitin interacting motifs of USP37 act on the proximal Ub of a di-Ub chain to enhance catalytic efficiency

Noah Manczyk, Gianluca Veggiani, Joan Teyra, Amy W. Strilchuk, Sachdev S. Sidhu*, Frank Sicheri*

Supplementary Figure S1

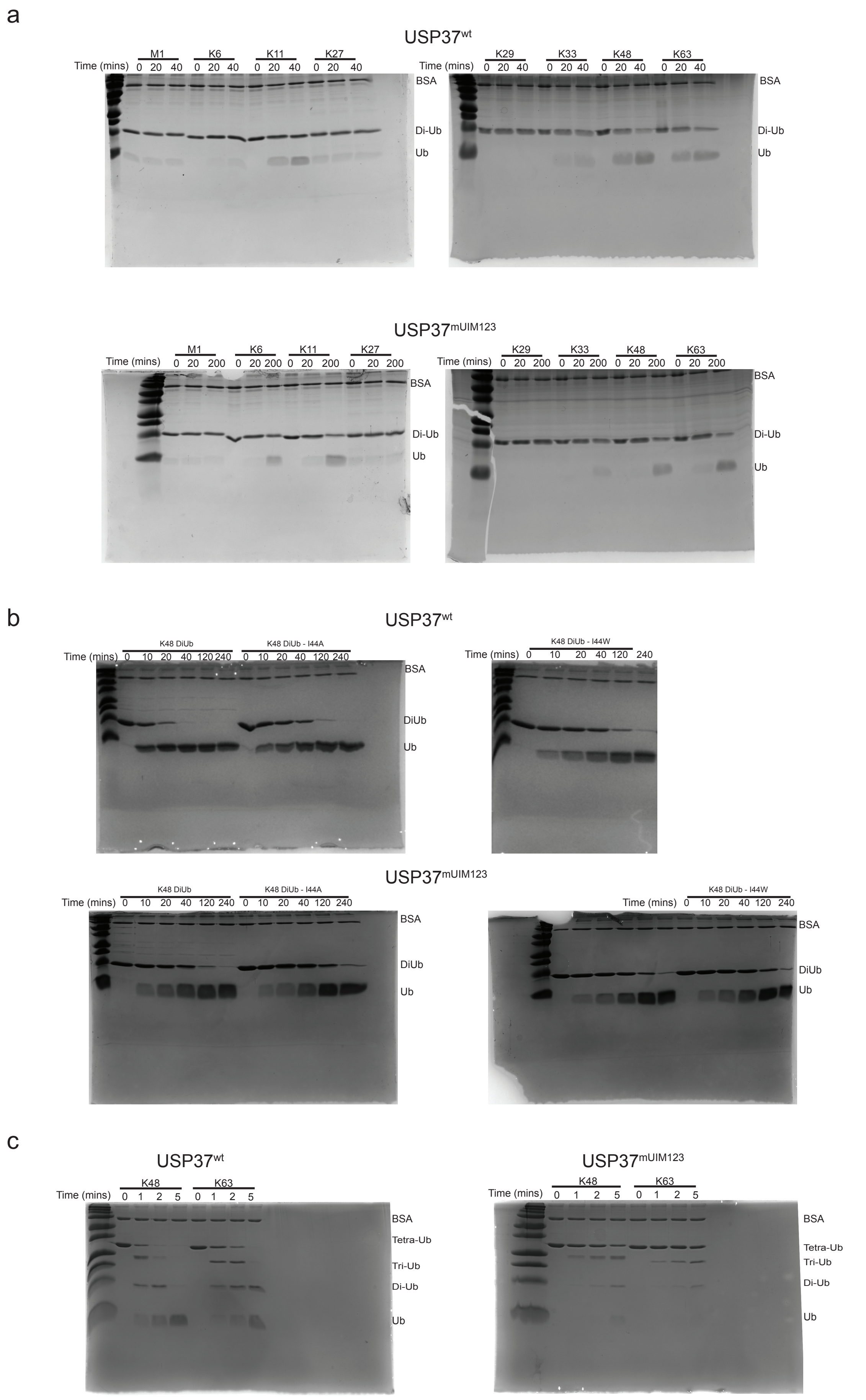

Supplementary Figure S1 - Continued

d

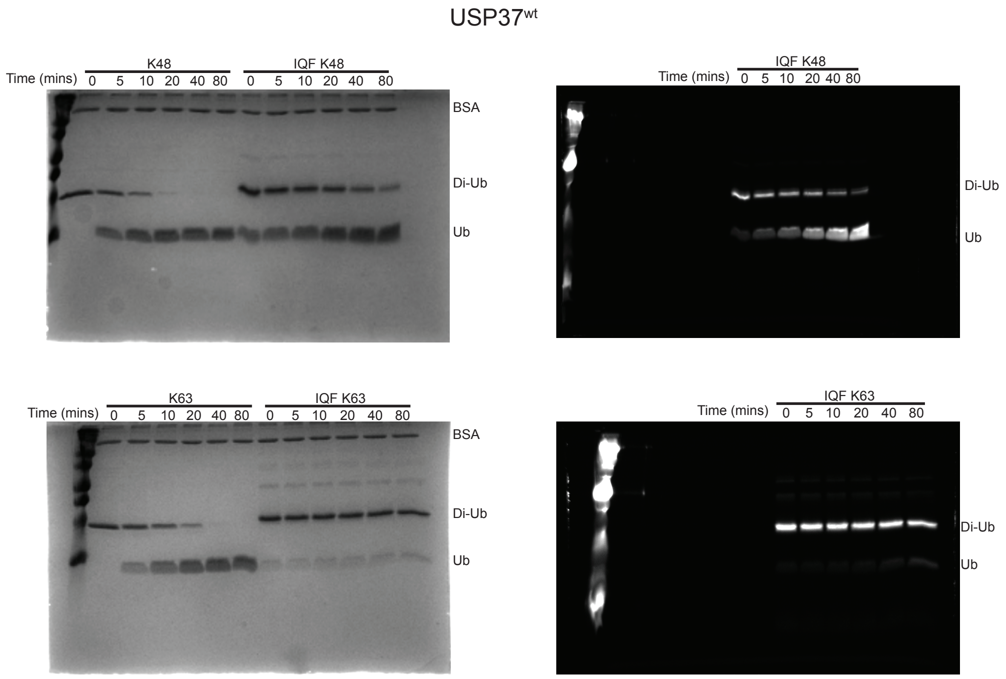

e

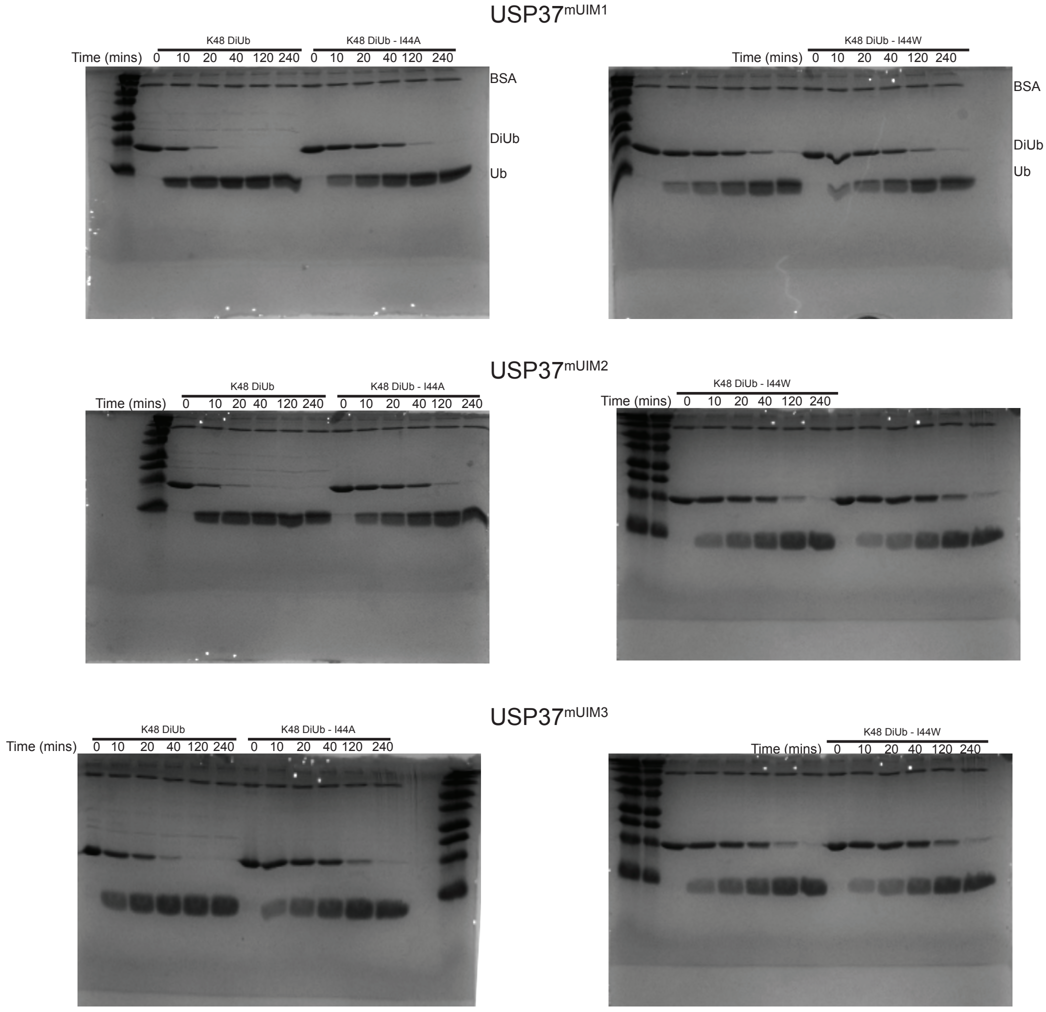

Supplemenetary Figure S1. Uncropped gel images. Uncropped images of gels depicted in: Figure 1b top and bottom panels (subpanel a), Figure 4b top and bottom panels (subpanel b), Figure S2 top and bottom panels (subpanel c), Figure S3 top and bottom panels (subpanel d), Figure S5 second, third, and fourth row of panels (subpanel e).

Supplementary Figure S2

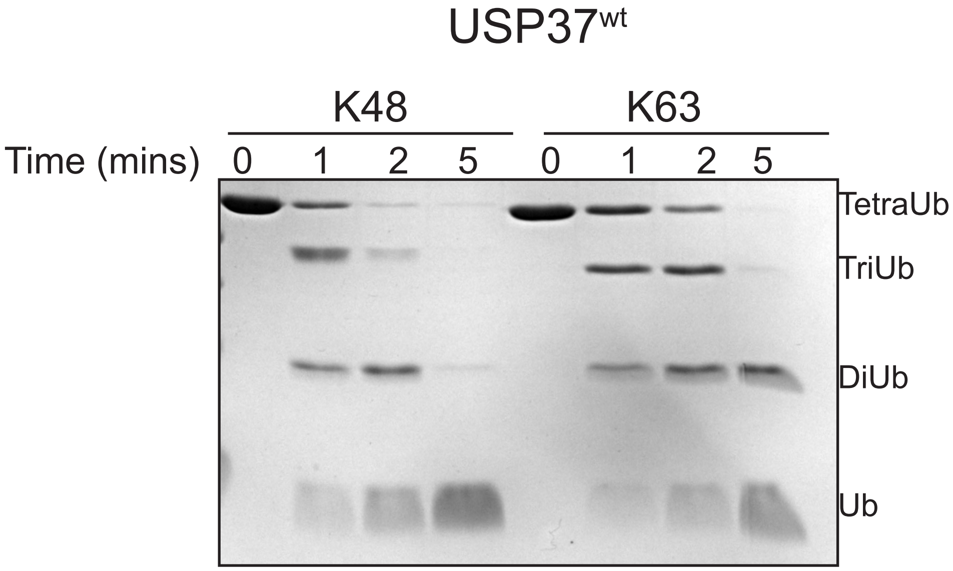

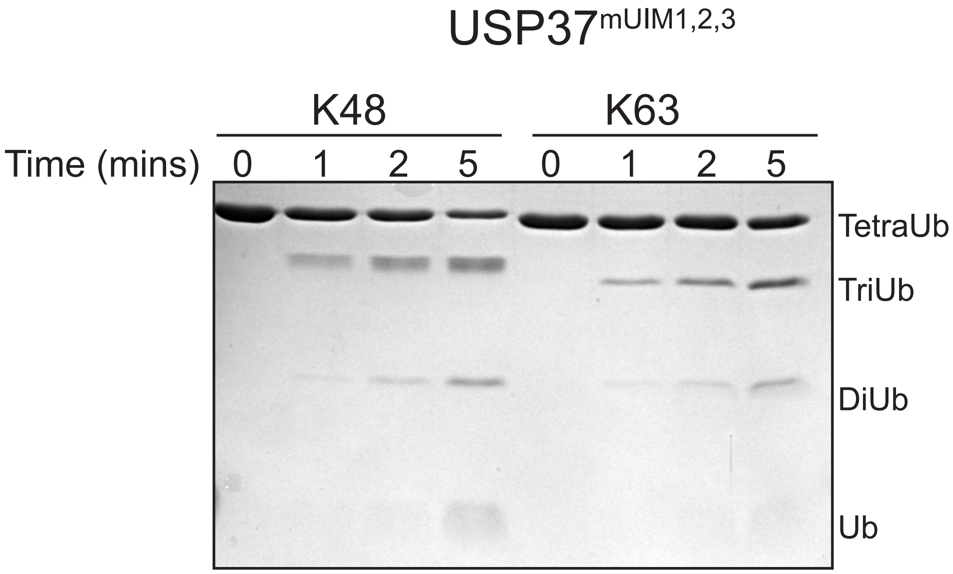

Supplementary Figure S2. K48 and K63 tetra-Ub cleavage specificity of USP37^{wt} and USP37^{mUIM1,2,3}. Deubiqutination time course for USP37^{wt} (top panel) and USP37^{mUIM1,2,3} (bottom panel) towards K48 and K63 tetra-Ub. Assay was performed in three independent experiments and representative gels are shown. USP37^{wt} and USP37^{mUIM1,2,3} were used at a concentration of 10 nM. Uncropped versions of gels are shown in Supplementary Figure S1.

Supplementary Figure S3

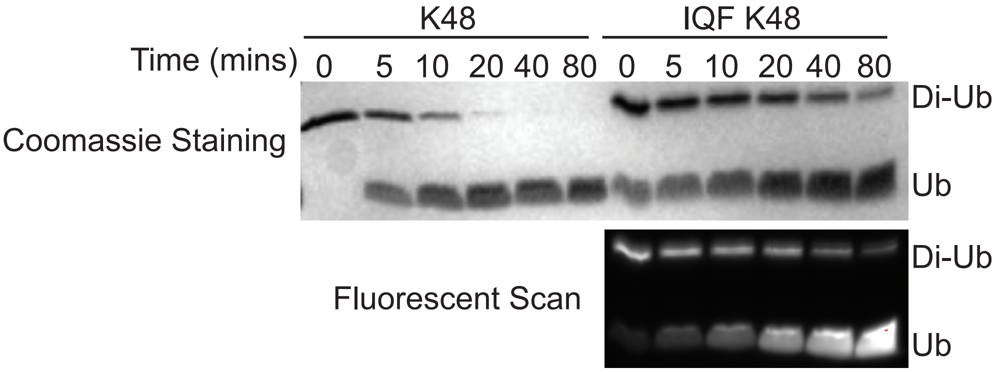

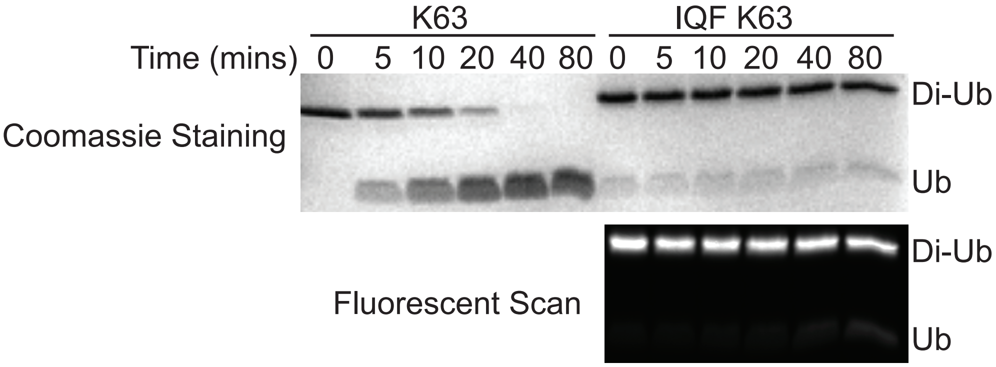

Supplementary Figure S3. USP37^{wt} activity towards unmodified and IQF modified K48 and K63 di-Ub chains. Time course for USP37^{wt} deubiquitination of K48 (top set of panels) and K63 (bottom set of panels) with unmodified and IQF modified di-Ub chains. Gels were fluorescently scanned to detect cleavage of IQF modified di-Ub chains and stained with Coomassie to detect cleavage of both unmodified and IQF modified di-Ub chains. Assay was performed in three independent experiments and representative gels are shown. USP37^{wt} and USP37^{mUIM1,2,3} were used at a concentration of 1 nM. Uncropped versions of gels are shown in Supplementary Figure S1.

Supplementary Figure S4

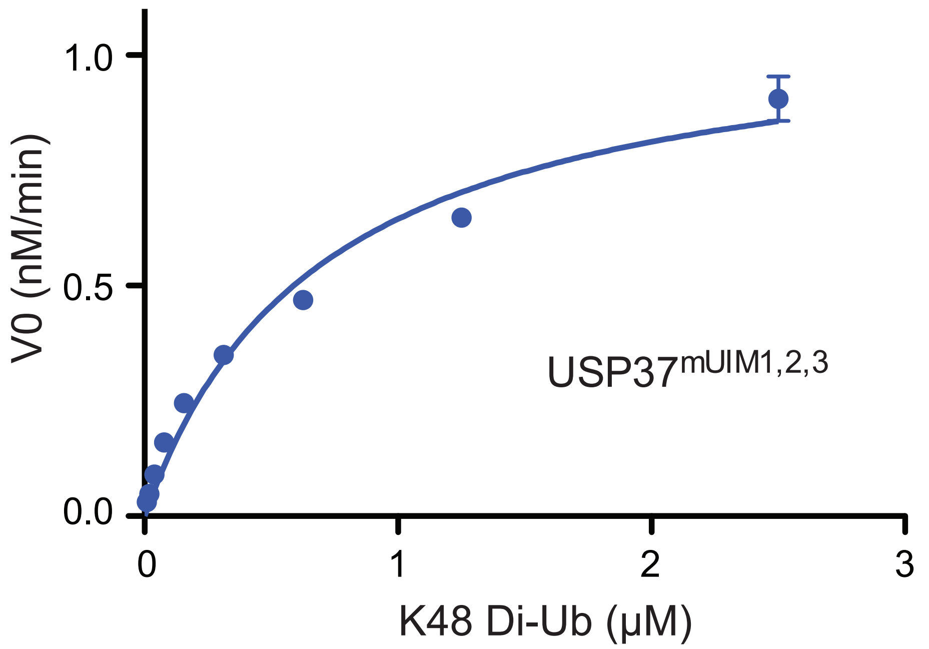

Supplementary Figure S4. Michaelis-Menten analysis of USP37^{mUIM1,2,3} for K48-linked IQF di-Ub. Curve represents measurements from three independent experiments measured in duplicate. Enzyme was used at 1.25 nM. The data for USP37^{mUIM1,2,3} was reproduced here from the K48 subpanel of Figure 2a for clarity.

Supplementary Figure S5

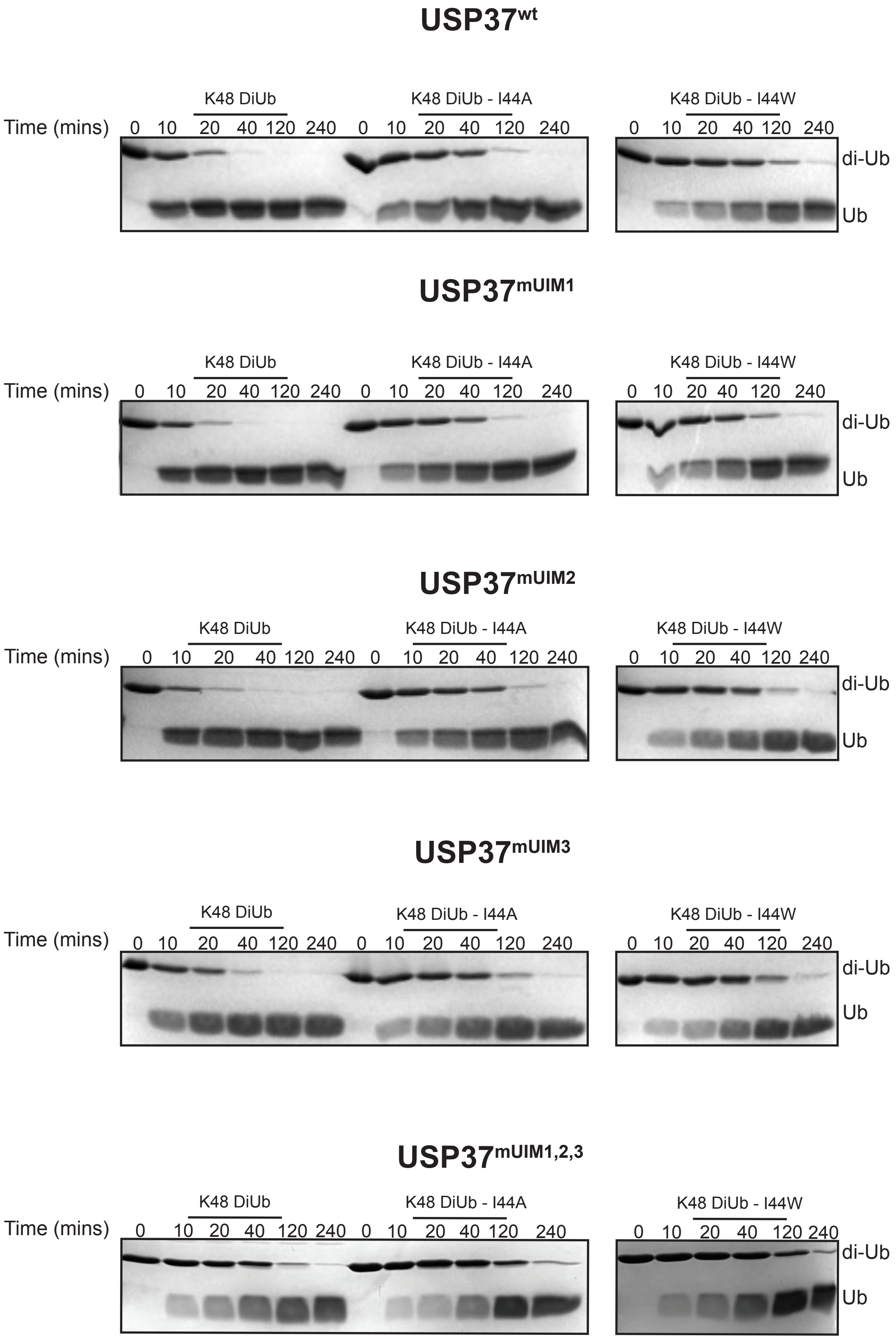

Supplementary Figure S5. Activity of USP37^{wt} and USP37 mutants towards proximally mutated K48 di-Ub. Deubiquitination time course for USP37^{wt}, USP37^{mUIM1}, USP37^{mUIM2}, USP37^{mUIM3}, and USP37^{mUIM1,2,3} towards K48, K48^{I44A}, and K48^{I44W} di-Ub. Assay was performed in three independent experiments and representative gels are shown. USP37 enzymes were used at a concentration of 5 nM. Black boxes delineate distinct gels or cropped areas of same gel. Uncropped versions of gels are shown in Figure S1B and S1E. The data for USP37^{wt} and USP37^{mUIM1,2,3} was reproduced here from Figure 4b for clarity.

Supplementary Figure S6

	1									10					15	45					50	60										70						76	76A	76B	76C
**Ub**	M	Q	I	F	V	K	T	L	T	G	K	T	I	T	L	F	A	G	K	Q	L	N	I	Q	K	E	S	T	L	H	L	V	L	R	L	R	G	G	-	-	-
**UbV.core**	-	-	-	-	-	-	-	-	-	-	-	-	-	-	-	-	-	-	-	-	-	-	-	N	-	L	-	F	-	-	-	-	M	-	-	L	S	-	S	R	
**UbV.UIM1**	-	-	-	-	-	N	-	-	-	A	M	P	-	-	-	-	-	-	S	L	-	-	-	R	-	N	-	S	-	Y	-	-	S	-	R	G	-	-	-	-	-
**UbV.UIM***	-	-	-	-	-	-	I	R	A	V	-	Y	-	N	-	-	-	-	M	-	-	-	-	K	-	K	-	I	-	-	V	-	-	-	H	-	S	L	R	A	G

Supplementary Figure S6. Sequence alignment of UbVs. Regions that were mutated in the phage-displayed libraries are shown and *dashes* denote the WT sequence.

Supplementary Figure S7

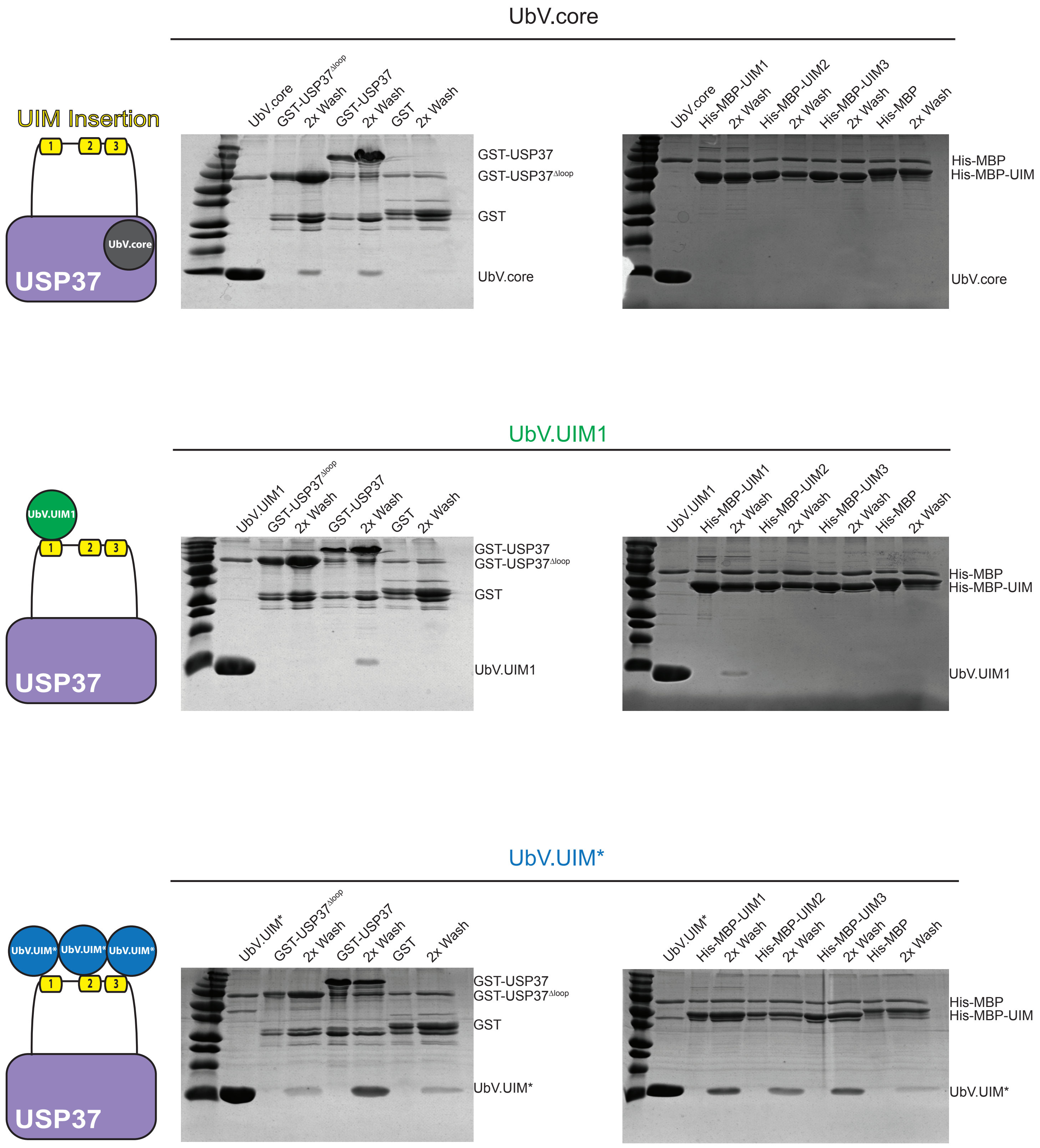

Supplementary Figure S7. Binding specificity of UbVs. UbV.core, UbV.UIM1 and UbV.UIM* were incubated with the indicated bait proteins. After washing, resulting fraction still bound to beads was analyzed by SDS-PAGE and Coomassie blue staining.

Supplementary Figure S8

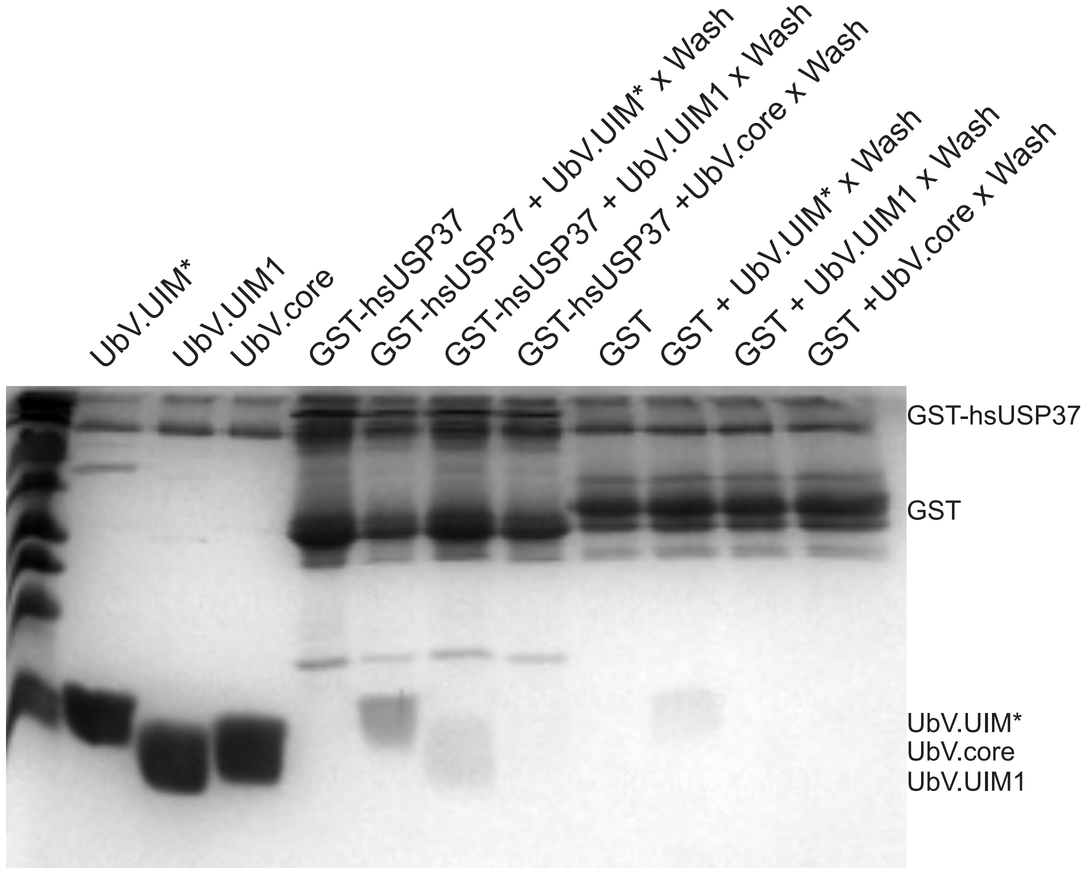

Supplementary Figure S8. UbV binding ability to human USP37. UbV.core, UbV.UIM1 and UbV.UIM* were incubated with human USP37 fused at the N-terminus to GST (GST-hsUSP37) and GST. After washing, resulting fraction still bound to beads was analyzed by SDS-PAGE and Coomassie blue staining.

Supplementary Figure S9

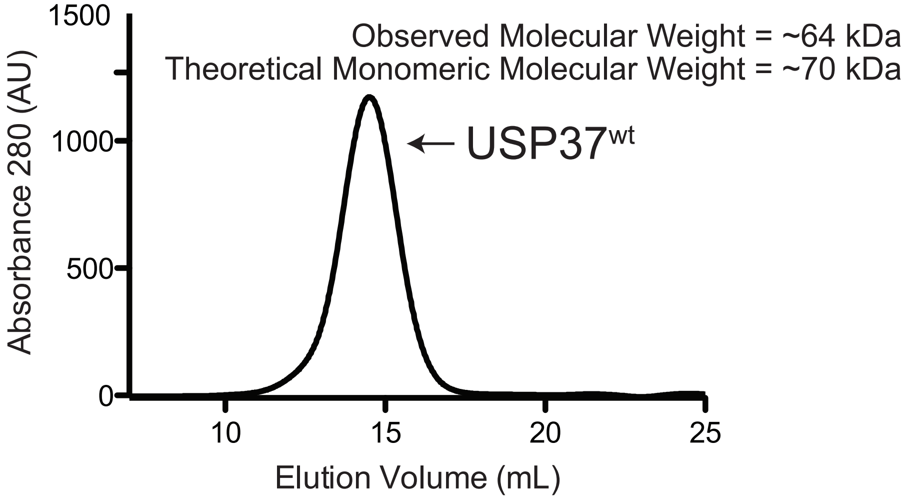

Supplementary Figure S9. USP37^{wt} is a monomeric protein. Size exclusion chromatography elution profile for USP37^{wt}.
